# Osteogenic effects of exosomes derived from human chorion membrane extracts

**DOI:** 10.1186/s40824-021-00218-6

**Published:** 2021-05-06

**Authors:** Yoon Young Go, Sung-won Chae, Jae-Jun Song

**Affiliations:** 1 Department of Otorhinolaryngology-Head and Neck Surgery, Korea University Guro Hospital, 80 Guro-dong, Guro-gu, Seoul, 08308 Republic of Korea; 2 Institute for Health Care Convergence Center, Korea University Guro Hospital, Seoul, 08308 Republic of Korea

**Keywords:** Exosome, Human chorion membrane extracts, Osteogenesis

## Abstract

**Objective:**

Human chorion membrane extracts (CME) are known to exhibit osteogenic effects when used for treating human osteoblast-like cells (MG63 cells), but the active compound in CME remains unknown. The aim of this study was to identify the presence of exosomes in CME and to determine the osteogenic effect of CME exosomes on MG63 cells.

**Methods:**

Exosomes were isolated from human placenta CME using the ExoQuick-TC solution and were characterized. The activity and deposition of alkaline phosphatase (ALP) on MG63 cells cultured with or without exosomes in osteogenic induction medium (OIM) were determined. Human amniotic membrane extracts (AME) were used as controls as they had not affected the osteogenic differentiation of MG63 cells in our previous study.

**Results:**

Transmission electron microscopy (TEM) revealed that exosomes isolated from CME and AME (CME-Exo and AME-Exo, respectively) had a cup-shaped structure. NanoSight™ particle tracking analysis (NTA) confirmed that the size of these exosomes was 100–150 nm. In vitro osteogenic experiments demonstrated that the exosomes from CME, but not those from AME, presented increased alkaline phosphatase (ALP) activity and resulted in the mineralization of MG63 cells in a dose-dependent manner.

**Conclusion:**

Exosomes were identified in CME and AME from the human placenta. Further, the exosomes from CME were found to be capable of promoting osteogenic differentiation, suggesting that exosomes are a key component of CME that stimulate the osteogenesis of human osteoblast-like cells. CME exosomes can be developed as promising therapeutic candidates for bone regeneration.

**Supplementary Information:**

The online version contains supplementary material available at 10.1186/s40824-021-00218-6.

## Introduction

The purpose of this study was to investigate the osteogenic effect of human chorion membrane exosomes (CME-Exo) using human osteoblast-like cells. Exosomes are small membrane-bound extracellular vesicles (EVs) with a diameter of approximately 30–150 nm [1, 2]. Exosomes are released from multi-vesicular bodies when vesicles fuse with the cell membrane and are produced in most cell types [1]. In addition, most body fluids, such as serum, saliva, urine, and bile, contain exosomes, which can be isolated using an in vitro experimental system [3]. It has been considered that exosomes play a key role in cell-cell communication, acting as a cell-cell mediators, by virtue of their cellular cargo, including proteins, lipids, mRNAs, micro RNAs, and lncRNAs [2, 4, 5]. Exosomes integrate into the recipient cells or target cells, affecting their fate (i.e., cell proliferation, differentiation, migration, and apoptosis) by regulating the inter-cellular signaling pathway [4, 6, 7].

In the human placenta, exosomes have been isolated from amniotic fluid or amniotic epithelial cells to determine the physiological and immunological state of the mother and baby during pregnancy [8–10]. Exosomes from human amniotic epithelial cells also have therapeutic functions, such as repairing lung injuries, inhibiting scar formation, and promoting wound healing, as well as in the field of obstetrics [11–13]. However, exosomes have not yet been isolated from the chorionic membrane of the human placenta.

Our previous studies had demonstrated that chorion membrane extracts (CME) from human placenta have an excellent osteogenic efficacy, especially with respect to the osteogenic differentiation of human mesenchymal stem cells (hMSCs) and osteoblast-like cells [14, 15]. However, the exact underlying mechanism and the key active compounds in CME that mediate osteogenesis in CME-treated osteoblasts are still unclear. Herein, we hypothesized that CME-Exo play an important role in mediating the osteogenic effect of CME in human osteoblast-like cells. To test our hypothesis, exosomes were isolated from human CME and their features were characterized. Then, the osteogenic effects of CME-Exo were then examined using osteoblast-like cells (MG63 cells). Osteogenesis is a multistep process, and can be divided into early, middle, and late stages [16, 17]. Treatment of MG63 cells with CME-Exo induced osteogenesis, ALP activity was critically increased from 3 to 7 days in the early stage of osteogenesis, and the mineral deposition was observed from 14 to 20 days in the late stage of osteogenesis [14, 18]. Therefore, we investigated the ALP activity and mineralization of CME-Exo-treated MG63 cells during osteogenesis in vitro.

## Materials and methods

### Isolation of exosomes from AME and CME

AME and CME were prepared as described in our previous studies [14, 15]. Human placentas were obtained from two donors with the approval from the Institutional Review Board (KUGH14239–002) of Korea University Guro Hospital. Extracts (AME and CME) from human placenta were mixed with the ExoQuick-TC™ solution (System Biosciences, USA) at a 1:5 ratio (extract: ExoQuick-TC solution), as described in the experimental procedure. Next, this mixture was incubated overnight at 4 °C. After centrifugation at 1500×*g* for 30 min, the supernatant was removed. The exosome pellet was resuspended in PBS and stored at − 80 °C.

### Transmission electron microscopy (TEM)

Isolated exosomes were resuspended in 1 mL of PBS, and 3 μL of the sample was adsorbed onto a glow-discharged copper grid covered with a carbon film. After 30 s, the samples were stained with 3% uranyl acetate and examined using a Tecnai T120 microscope. Images were acquired at 67,000× magnification using an FEI Eagle 4 K × 4 K CCD camera. The size of all exosomes was measured.

### Nanoparticle tracking analysis (NTA)

The size and concentration of the isolated exosomes were analyzed using a NanoSight™ LM10-HS10 system (NanoSight Amesbury, UK). Each exosome sample was diluted with exosome-depleted fetal bovine serum (FBS) to obtain a measurable concentration. A monochromatic laser beam (405 nm) in combination with NanoSight™ tracking software version 2.3 was used to analyze the diluted exosomes.

### Alkaline phosphatase (ALP) assay

MG63 cells were cultured in growth medium (GM) (DMEM supplemented with 10% fetal bovine serum (Gibco, USA) and 100 U/mL penicillin/streptomycin (Gibco)). Osteogenic induction medium (OIM) consisting of 10 nM dexamethasone (Sigma, USA), 0.2 mM ascorbic acid (Sigma), and 10 mM β-glycerophosphate (Sigma) in GM was used for the in vitro osteogenic differentiation. OIM with or without the extracts was replaced every 2–3 days. After 7 days of culture in OIM with or without AME or CME, the ALP activity was measured in hMSCs using a SensoLyte® *p*NPP Alkaline Phosphatase Assay Kit (ANASPEC, USA) according to the supplier’s protocol. ALP activity was determined using a microplate reader at a wavelength of 405 nm.

### Alizarin red S staining

After 14 days of in vitro osteogenesis, the cells were washed with PBS and fixed in 4% paraformaldehyde for 15 min at room temperature. After fixation, cells were washed twice with PBS, and then staining was performed by treating with Alizarin red (Millipore, Germany) for 20 min at room temperature. Finally, the cells were washed three times with distilled water, the color change was registered, and then 100 mM cetylpyridinium chloride (Sigma) was added for the de-staining. The rate of mineralization was quantified using a microplate reader at 570 nm.

### Statistical analysis

An independent two-sample test of AME and CME was used in this experiment. A *t-*test was used to assess the differences in continuous variables between the two independent groups. All experiments were performed at least in triplicates, and the data are expressed as mean ± standard deviation (SD). A *p*-value < 0.05 was considered significant. Statistical analyses were performed using SPSS 20 for Windows (SPSS, USA).

## Results

**Fig. 1:**
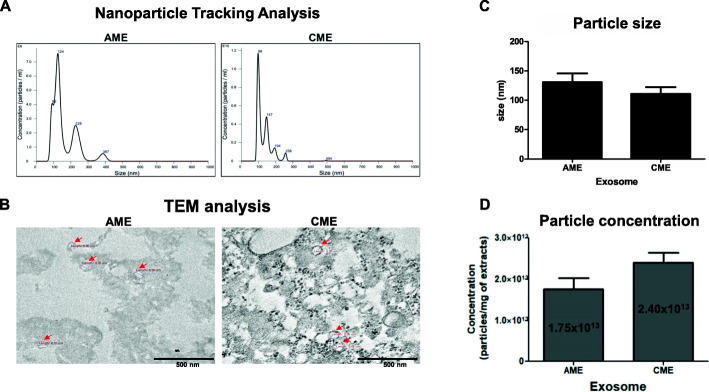
Characterization of exosomes from human amnion and chorion membrane extracts. **a** Nanoparticle tracking analysis (NTA) of AME-Exo and CME-Exo. **b** Transmission electron microscopy (TEM) image of AME-Exo and CME-Exo morphology. **c** Average diameter of AME-Exo and CME-Exo. **d** Particle concentration of AME-Exo and CME-Exo. The values indicate the number of exosomes in 1 mg of each extract

The exosomes isolated from human AME and CME using the ExoQuick-TC™ solution were first characterized based on size. NTA revealed that the exosomes ranged in size from 80 to 400 nm for AME and from 90 to 260 nm for CME. The appropriate size of exosomes (50–130 nm) is included in these results, but many macrovesicles were also observed in AME and CME (Fig. 1a). Next, we analyzed the exosome shape using TEM, and a cup-shaped structure was observed in both AME and CME (Fig. 1b and Supplementary Figure 1). NTA revealed that the average size of the AME and CME exosomes was in the range of 100–150 nm (Fig. 1c). We also determined the concentration of exosomes obtained from AME and CME. The concentration of exosomes obtained from CME was higher than that of exosomes from AME, but the difference was not significant (Fig. 1d). These results indicated that exosomes isolated from human AME and CME exhibited physical characteristics similar to those of other exosomes [19, 20]. Therefore, we decided to use exosomes from human amniotic membrane extracts in further osteogenic studies.

**Fig. 2:**
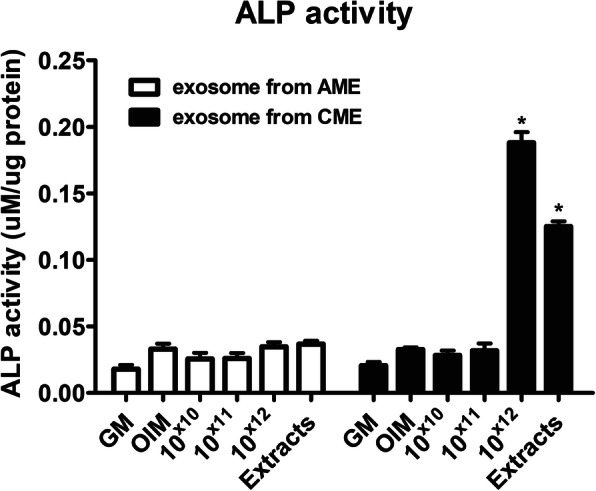
Effect of CME-Exo on ALP activity in MG63 cells. ALP activity was determined in MG63 cells after 7 days of culture with OIM in the presence or absence of AME-Exo and CME-Exo. Extracts indicate AME (white bar) and CME (black bar). Each extract was used at a concentration of 200 μg/mL (control). Error bars indicate the mean and SD; *p < 0.05 compared with the OIM control

**Fig. 3:**
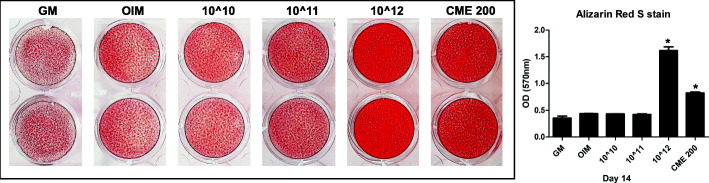
Effect of CME-Exo on the mineralization of MG63 cells. Alizarin Red S staining was performed in MG63 cells cultured with OIM in the presence or absence of CME-Exo after 14 days of in vitro osteogenic induction. CME 200: 200 μg/mL CME (control). Samples were de-stained, and then the absorbance was measured at 570 nm. Error bars indicate the mean and SD; *p < 0.05 compared with the OIM control

To investigate the osteogenic efficacy of exosomes from AME and CME, we examined ALP activity in the presence or absence of AME-Exo and CME-Exo during in vitro osteogenesis of MG63 cells. Several concentrations of exosomes were used and 200 μg/mL AME and CME extracts were used as positive controls. We had previously demonstrated that CME—but not AME—significantly increased the ALP activity in MG63 cells on day 7 [14]. Consistently, AME-Exo and AME did not elevate the ALP activity of MG63 cells, whereas a stimulatory effect of CME-Exo and CME on ALP activity was observed. A high concentration of CME-Exo (1 × 10^12^ particles/mL) and CME resulted in a significant increase in ALP activity (Fig. 2). The cellular toxicity of CME-Exo was not observed at day 7 (Supplementary Figure 2). After 14 days of in vitro osteogenic differentiation, cells were stained with Alizarin Red S to investigate the mineralization of the extracellular matrix on osteoblast-like cells. The distinct color change observed in 1 × 10^12^ CME-Exo particles and CME-treated cells indicated an increase in mineralization during the in vitro osteogenesis of MG63 cells. No significant difference in the low concentration of CME-Exo was observed in this experiment. Similar to the ALP results, the rate of mineralization of MG63 cells in the presence of 1 × 10^12^ CME-Exo was higher than that of CME-treated cells (Fig. 3). These results clearly showed that exogenous exosomes (1 × 10^12^ particles/mL) from CME promoted ALP activity and the mineralization of MG63 cells, indicating that CME exhibits osteogenic effects when used to treat human osteoblast-like cells.

## Discussion

We first demonstrated the functional property of the human chorion membrane in the placenta, which is usually discarded after delivery [15]. The human placenta contains numerous biological molecules, such as growth factors, cytokines, and bioactive proteins (i.e., collagen, fibronectin, laminin, and proteoglycans). It is an ideal tissue for natural biomaterials in the field of tissue engineering and regeneration medicine [21]. Therefore, the amniotic membrane in the placenta has been studied by many researchers and applied to the wound as a biological bandage [21–23]. In contrast, the chorion membrane in the placenta has not been studied and used in the clinical field because of its structure. The chorion membrane is thick and opaque, being a complex combination of three cellular layers, whereas the amniotic membrane is thin, transparent, and composed of simply layered amniotic-epithelial cells. Compared with the amniotic membrane, the chorion membrane could not be used as a scaffold for wound healing, even though it contains 4–5 times more bioactive components than the amniotic membrane [24]. We developed a human chorion membrane in an extract form and then used it for regenerating the bone, which resulted in promoting the effects of CME on osteoblast osteogenesis [14]. However, their clinical usefulness as therapeutic drugs for bone formation is limited by their complexity. A clinical drug in an extract form is difficult to develop due to quality control. Further, there are established protocols, many reports and data, and a good manufacturing practice (GMP) system for clinical trials aimed at the development of exosomes [25]. Therefore, we isolated exosomes from CME and then confirmed their osteogenic efficacy using osteoblasts.

Exosome therapy is currently a novel approach for osteogenesis in the clinic [26]. It is still unclear how we can regulate bone homeostasis using exosomes [27]. Recently, several miRNAs in MSC-derived exosomes (upregulation: miR-199b, miR-218, miR-203, and miR-302b; downregulation: miR-221, miR-155, miR-885-5p, and miR-181a) have been studied as osteogenic regulators during MSC osteogenic differentiation. miRNA is a functional cargo in exosomes, and it plays an important role as a key modulator in promoting or reducing osteoblast osteogenesis [28]. In addition, exosomes derived from immune cells, such as dendritic cells and monocytes, influence osteogenesis [29, 30].

In this study, CME-Exo significantly elevated the osteogenesis of MG63 cells, showing their potential as positive regulators of osteogenesis in osteoblasts. Although the exact mechanism underlying the osteogenic effect of CME-Exo is not known, CME-Exo can be developed as a therapeutic for bone regeneration. CME-Exo provides a stimulatory environment in which MG63 cells can rapidly undergo differentiation, i.e., osteogenesis is observed in vitro.

## Conclusion

In this study, we investigated the osteogenic effects of CME-Exo using human osteoblast-like cells. Exogenous treatment with CME-Exo resulted in significantly increased the ALP activity and mineral deposition in MG63 cells during osteogenesis. Taken together, our study demonstrates the osteogenic effects of exosomes in CME in the context of human osteoblast-like cells.

## Supplementary Information

**Additional file 1: Supplementary Figure 1.** The magnified TEM images of AME-Exo and CME-Exo. The cup-shaped structure of AME and CME exosomes were determined by TEM. The scale bar represents 500 nm.

**Additional file 2: Supplementary Figure 2.** The morphology of CME-Exo treated MG63 cells. The morphology of GM, OIM and CME-Exo treated MG63 cells visualized by light microscopy at day 7. The scale bar represents 500 nm.

## Data Availability

All data analyzed from this study are included in this published article.

## Abbreviations

CME: Chorion membrane extracts
AME: Amniotic membrane extracts
hMSCs: Human mesenchymal cells
GM: Growth medium
OIM: Osteogenic induction medium
TEM: Transmission electron microscopy
NTA: Nanoparticle tracking analysis
