# Supplementary figures and images for: Osteogenic effects of exosomes derived from human chorion membrane extracts
Source: Biomater Res. 2021 May 6;25:16. doi: 10.1186/s40824-021-00218-6 (PMC8101178; doi:10.1186/s40824-021-00218-6)

# TEM analysis

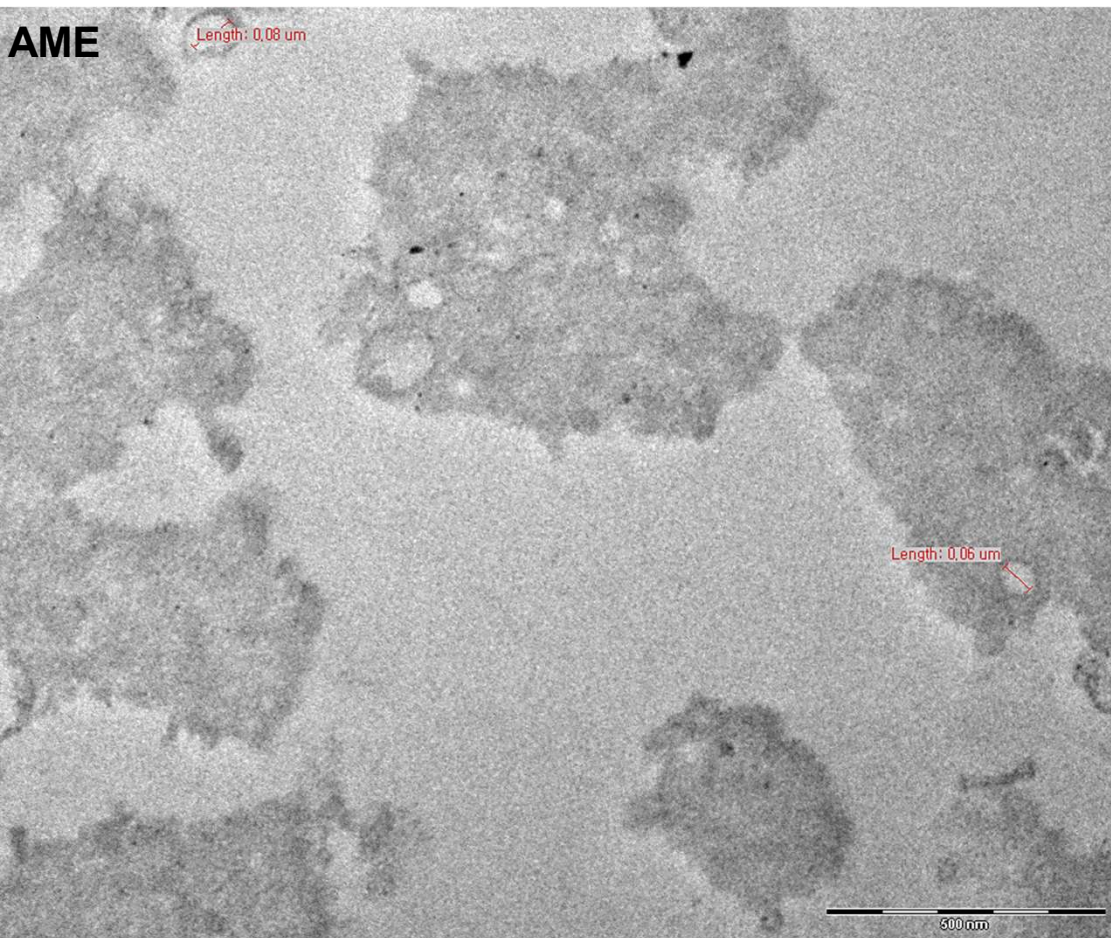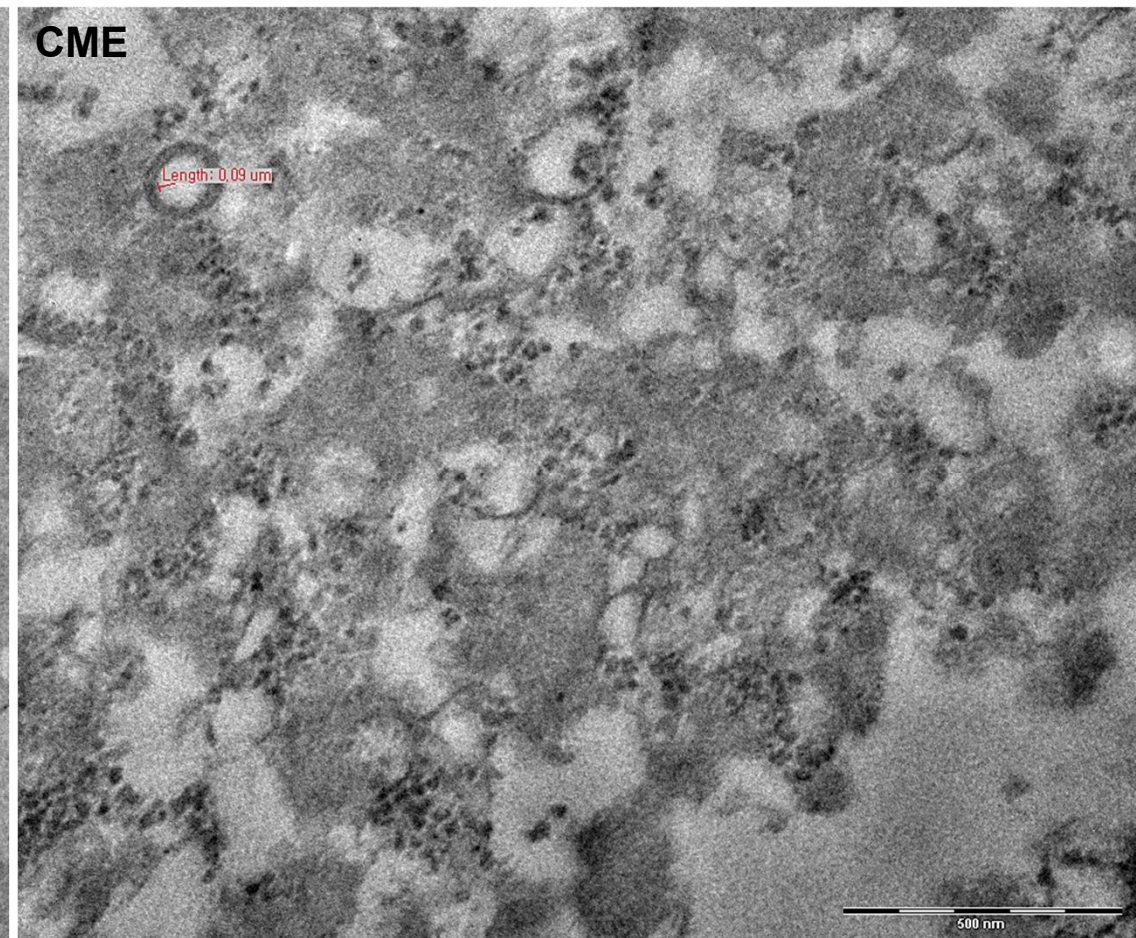

Supplement: Supplementary file 1 — Additional file 1: Supplementary Figure 1. The magnified TEM images of AME-Exo and CME-Exo. The cup-shaped structure of AME and CME exosomes were determined by TEM. The scale bar represents 500 nm. [file 40824_2021_218_MOESM1_ESM.pdf]

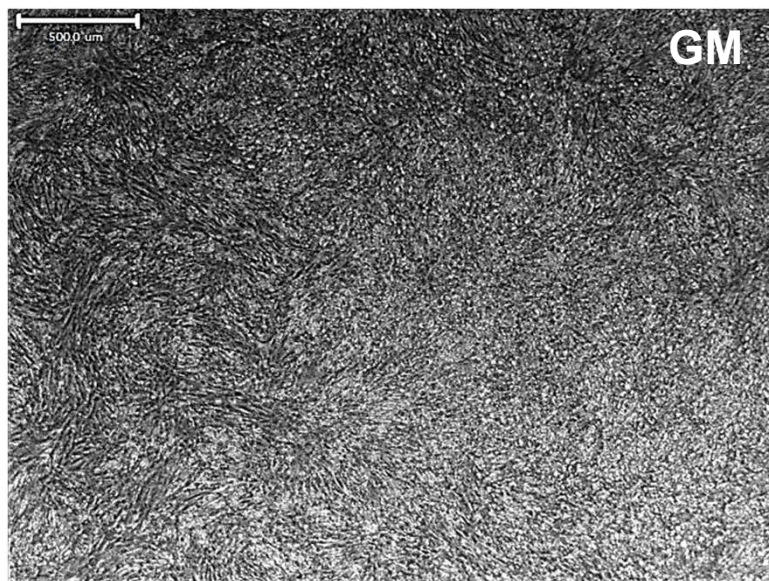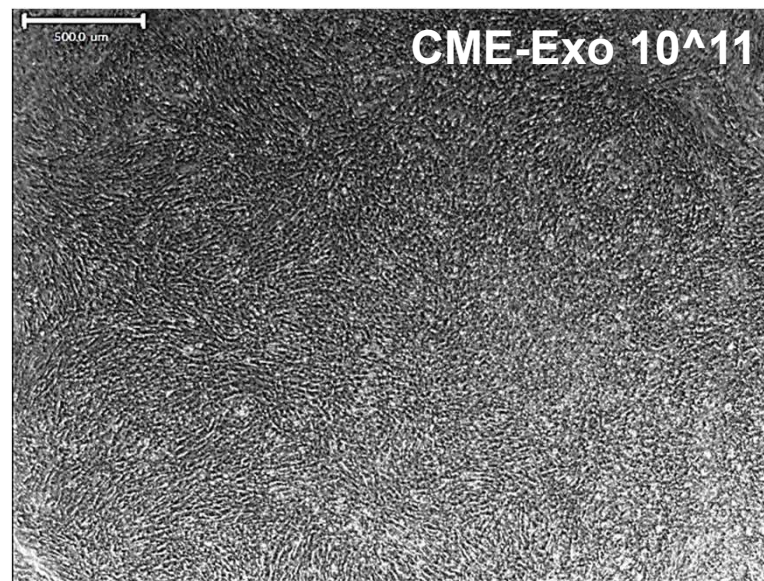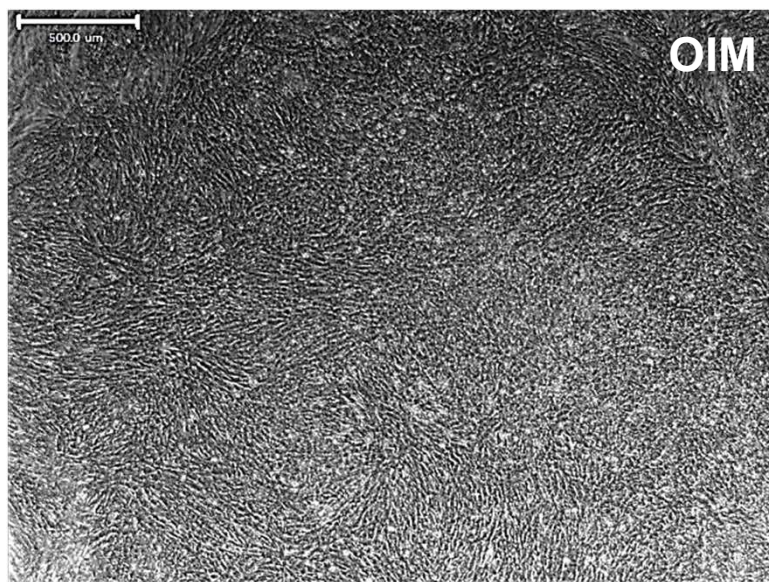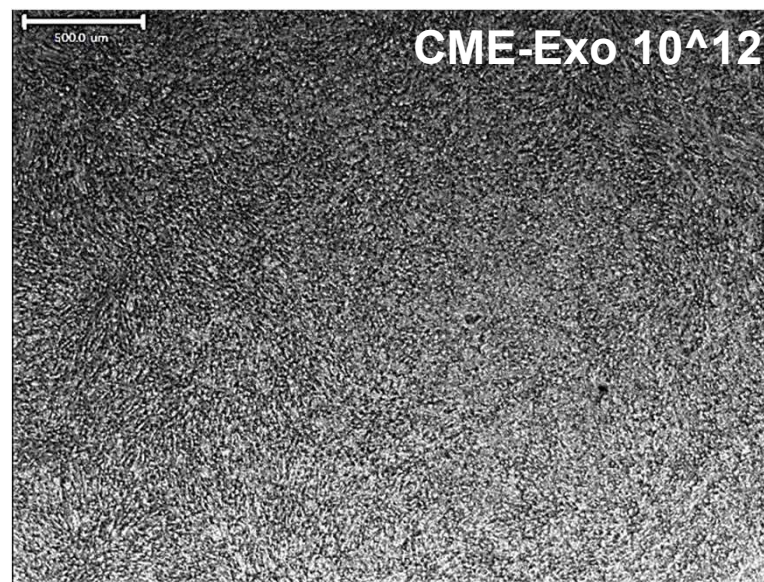

Day 7

Supplement: Supplementary file 2 — Additional file 2: Supplementary Figure 2. The morphology of CME-Exo treated MG63 cells. The morphology of GM, OIM and CME-Exo treated MG63 cells visualized by light microscopy at day 7. The scale bar represents 500 nm. [file 40824_2021_218_MOESM2_ESM.pdf]
